# Infectious Aortitis with Abdominal Aortic Aneurysm in a 47-Year-Old Female with Systemic Lupus Erythematosus

**DOI:** 10.1155/2019/4532169

**Published:** 2019-04-08

**Authors:** Valerie R. Ramiro, Carmegie C. Saliba, John Anthony D. Tindoc, Marinette R. Jambaro, Enrique M. Chua, Donna Ricca M. Hornilla, Maria Teresa B. Abola

**Affiliations:** ^1^Section of Cardiology, University of the Philippines–Philippine General Hospital, Manila, Philippines; ^2^Department of Medicine, University of the Philippines–Philippine General Hospital, Manila, Philippines; ^3^Department of Pathology, University of the Philippines–Philippine General Hospital, Manila, Philippines; ^4^Section of Rheumatology, University of the Philippines–Philippine General Hospital, Manila, Philippines; ^5^Section of Thoracic Cardiovascular Surgery, University of the Philippines–Philippine General Hospital, Manila, Philippines

## Abstract

Aortic aneurysms are not commonly reported among patients with systemic lupus erythematosus (SLE). We report a case of a 47-year-old Filipino female diagnosed with SLE 17 years ago maintained on prolonged oral steroids, azathioprine, and hydroxychloroquine. She also had lupus nephritis, secondary hypertension, and dyslipidemia. She initially presented with a week-long watery nonbloody diarrhea with associated diffuse crampy abdominal pain and generalized weakness. She was admitted for a week at a provincial hospital and was given an unrecalled antibiotic with resolution of symptoms. Upon discharge, however, she experienced two weeks of severe right lower quadrant pain radiating to the back and left lower quadrant, with no history of diarrhea, vomiting, dysuria, and fever. Complete blood count showed slight leukocytosis and elevated C-reactive protein. Abdominal imaging revealed a saccular infrarenal aneurysm with dissection. An atherosclerotic mechanism was primarily considered, but a vasculitic process was likewise considered due to elevated acute phase reactants. The initial plan was Endovascular Aneurysm Repair (EVAR) but due to financial limitations, an exploratory laparotomy with infrarenal endoaneurysmorrhaphy was eventually performed. Intraoperative findings were a saccular infrarenal aneurysm with dissection up to the proximal right common iliac artery and an abscess compartment within the false lumen in the anterior aortic wall. Abscess culture yielded high growth of *Salmonella* group B. Micrographs of the aortic wall biopsy showed fibrin deposition necrosis and calcification with peripheral viable cellular infiltrates consisting of neutrophils and foamy macrophages. Inadvertently placing an endovascular graft in an infected aortic aneurysm would have led to graft infection and catastrophic morbidity. We highlight the significance of having a high index of suspicion for infectious causes of aortitis among immunocompromised patients presenting with aneurysm prior to pursuing an endovascular versus an open approach for repair.

## 1. Introduction

Systemic lupus erythematosus (SLE) is an autoimmune disease with a multifaceted pathophysiology and multiorgan involvement. Cardiovascular disease is a major cause of morbidity and mortality complicating SLE [1]. However, diseases of the aorta, particularly aneurysms and dissections, among SLE patients are uncommonly reported [2].

## 2. Case

We report a case of a 47-year-old Filipino female diagnosed with SLE 17 years ago maintained on prolonged oral prednisone 10 mg/day, azathioprine, and hydroxychloroquine. She also had chronic kidney disease from lupus nephritis, secondary hypertension, and dyslipidemia. She was a nonsmoker.

She initially presented with a week-long watery nonbloody diarrhea with associated diffuse crampy abdominal pain and generalized weakness. There was no fever nor vomiting. She was admitted for a week at a provincial hospital and was given an unrecalled antibiotic with resolution of symptoms. Upon discharge, however, she experienced severe right lower quadrant pain radiating to the back and left lower quadrant for two weeks, with no history of diarrhea, vomiting, dysuria, and fever. She was readmitted at the provincial hospital where diagnostics revealed anemia and urinary tract infection, for which she was transfused with packed red blood cell units and given unrecalled intravenous antibiotics, respectively. Blood cultures were initially negative. Abdominal imaging revealed bilateral renal parenchymal disease and an infrarenal aortic aneurysm. Appendicitis was ruled out by symptomatology and imaging. She was then transferred to our institution for surgical repair of the aneurysm.

**Figure 1:**
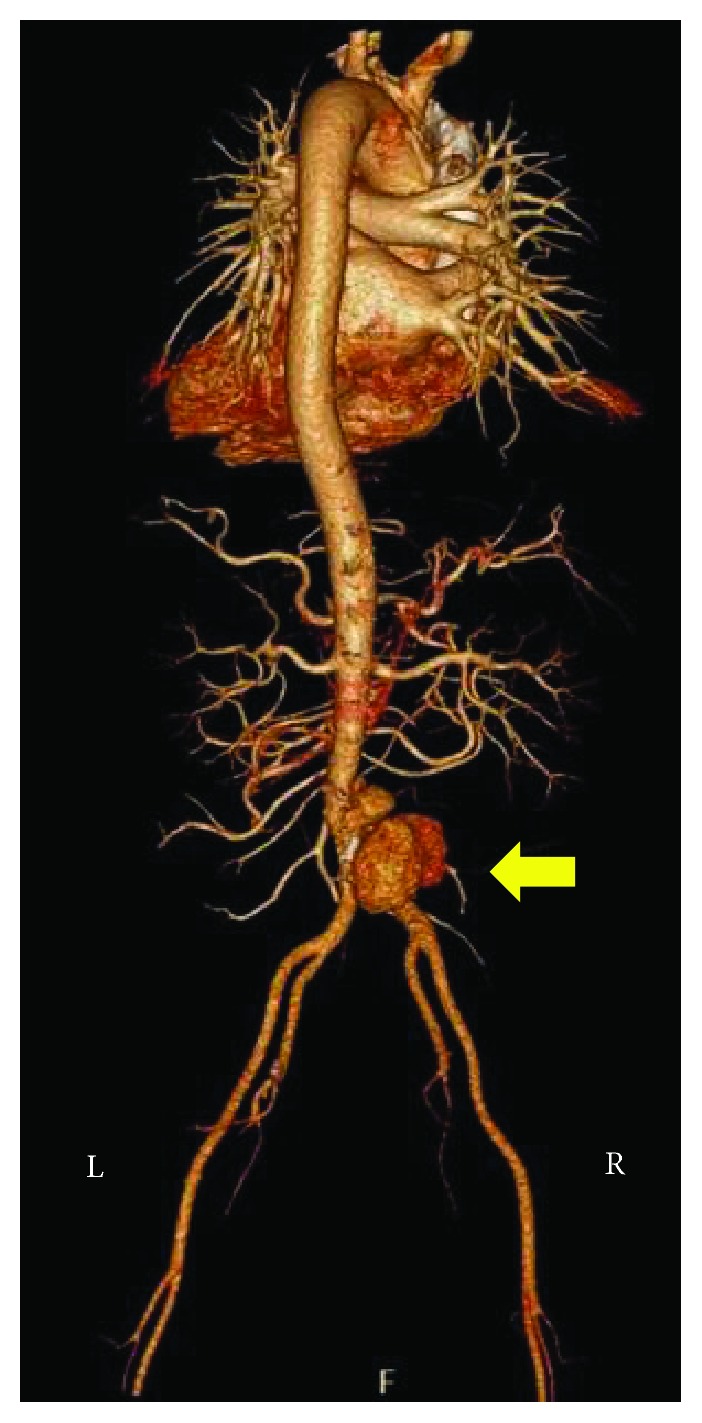
This is a 3-dimensional reconstruction of the CT aortogram, with the arrow indicating the saccular component of the aneurysm.

**Figure 2:**
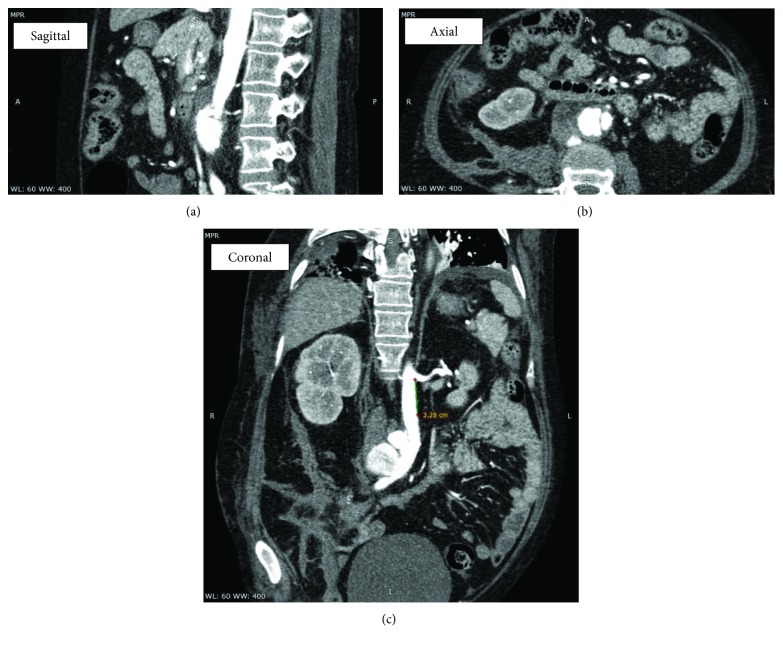
Multiplanar images demonstrating the infrarenal abdominal aorta. The dissection is seen arising 3.3 cm distal to the take-off of the left renal artery and extending to the iliac bifurcation, with an involved length of 7 cm.

**Figure 3:**
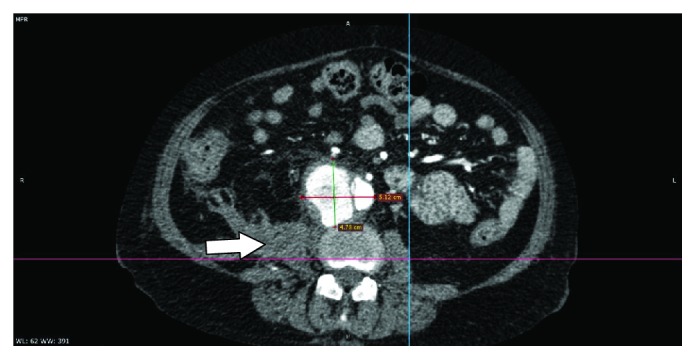
This is an axial view at the level of L4, demonstrating the saccular aneurysm at its maximal caliber of 5.1 × 4.8 cm (W × AP). An intimal flap is seen along the right lateral aspect of this dilatation. The saccular component of the aneurysm arises from the false lumen and is oriented anterolaterally to the right. No definite perivascular contrast extravasation is appreciated indicating that the point of rupture has likely been walled off. A heterogenous, predominantly hypodense fluid collection exhibiting minimal peripheral enhancement is appreciated at the right retroperitoneum, probably subacute hematoma (arrow).

During her admission at the surgical ward, antihypertensive medications were titrated to keep her blood pressures less than 120/80. Prednisone was given at 1 mg/kg/day. Hydroxychloroquine 200 mg OD, mycophenolate mofetil 500 mg BID, and atorvastatin 40 mg OD were continued. She continued to have intermittent abdominal pain. There was no fever, overt bleeding, dysuria, or recurrence of diarrhea. Complete blood count showed slight leukocytosis, and the C-reactive protein was elevated. A computed tomography (CT) aortogram revealed an infrarenal aneurysm with signs of dissection and retroperitoneal hematoma formation, indicative of leakage (see Figures 1–3). Given the absence of fever and no signs of ongoing infection, antibiotics were not yet started. An atherosclerotic mechanism was primarily considered, but a vasculitic process was likewise considered due to elevated acute phase reactants.

**Figure 4:**
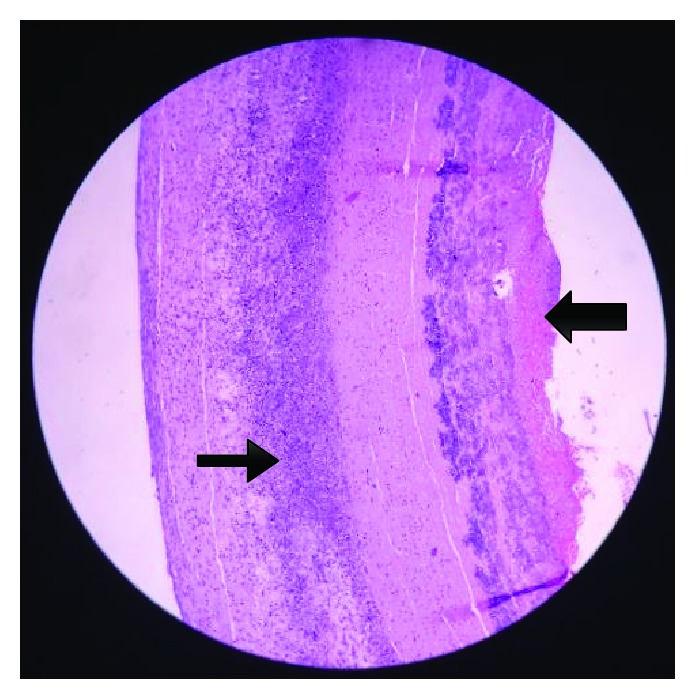
This is a section of the vascular wall adjacent to the main thrombus. Neutrophilic infiltrates (thin arrow) can be appreciated within the layers of the wall. At the intimal side, there is fibrin deposition (thick arrow) indicating an endothelial state favorable towards coagulation.

**Figure 5:**
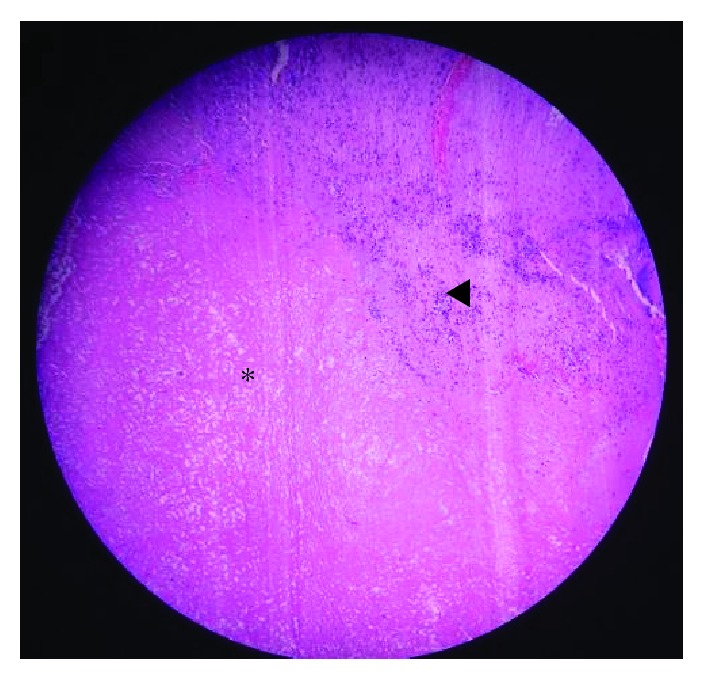
This is a section taken from the main thrombus showing heavy deposition of eosinophilic fibrin material (∗) with entrapped erythrocytes and inflammatory cells (◀).

**Figure 6:**
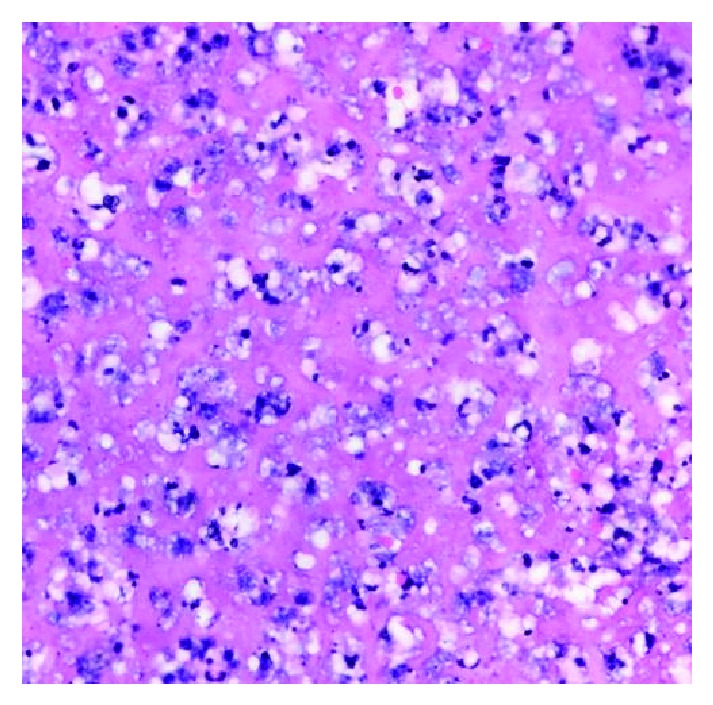
At the periphery of the thrombus with viable cellular material, clusters of foamy macrophages or foam cells can be seen.

The initial plan was Endovascular Aneurysm Repair (EVAR) but due to financial limitations, an exploratory laparotomy with infrarenal endoaneurysmorrhaphy was eventually performed. Intraoperative findings were a saccular infrarenal aneurysm with dissection up to the proximal right common iliac artery and an abscess compartment, with an aspirated volume of approximately 5 mL, within the false lumen in the anterior aortic wall. The entire infected aneurysmal segment was resected, and piperacillin-tazobactam was immediately started. Abscess culture yielded a high growth of *Salmonella* group B. Guided by the sensitivity pattern, the antibiotic was shifted to Ceftriaxone. This was continued after discharge as outpatient parenteral antibiotic therapy to complete 6 weeks then a lifetime of chronic suppressive therapy with trimethoprim-sulfamethoxazole (TMP-SMX) 160/800 mg OD. Syphilis and HIV screening were both negative. Micrographs of the aortic wall biopsy showed fibrin deposition necrosis and calcification with peripheral viable cellular infiltrates consisting of neutrophils and foamy macrophages consistent with an atherosclerotic process (see Figures 4–6). Given the histopathologic findings that favored aortitis rather than vasculitis, steroids were tapered down gradually. Aspirin was started postoperatively. Atorvastatin and antihypertensive medications were continued. She was discharged after 2 weeks and followed up regularly at the outpatient clinic.

## 3. Discussion

The etiology of SLE remains unknown but is likely multifactorial from genetic, immunologic, and environmental mechanisms. It occurs more commonly among women of reproductive age. The prevalence rate in the United States is around 20 to 150 per 100,000 women [3]. Among Asian countries, the prevalence rate falls within 30-50/100,000 people [4]. Aortic involvement has been found among many rheumatologic conditions and may present with a plethora of clinical manifestations [5]. Aortic diseases in SLE are not commonly reported. However, a case-control study found that SLE patients have a higher proportion of aortic aneurysms compared with age- and sex-matched controls, with an odds ratio of 4.5 [6].

Several pathophysiologic hypotheses have been purported to explain the occurrence of aortic aneurysms in SLE. Popular theories include an autoimmune process, such as vasculitis with associated medial degeneration, or an atherosclerotic process due to longstanding steroid use, hypertension, and dyslipidemia [2, 7]. Transforming growth factor *β*1 (TGF-*β*1) is a suppressor of vascular wall inflammation as well as inhibitor of smooth muscle proliferation; type I interferon is associated with impaired endothelial function. Downregulation of TGF-*β*1 and activation of interferons in SLE have been suggested as immunologic mechanisms contributing to arterial wall dysfunction [6]. A meta-analysis conducted by Kurata and colleagues in 2011 examined the pathophysiological relationship of certain clinical and histopathologic findings to the formation of aortic aneurysms in SLE. They included 35 cases reported over the past 40 years of SLE with aortic aneurysms, including those complicated by dissection. The study found that aortic involvement in SLE affected relatively younger individuals, with an average age of 44.5 years, and that the thoracic aorta was more commonly involved. This is contrary to aneurysms found in the general population which are usually discovered in the sixth decade of life and more commonly affecting the abdominal rather than the thoracic segments. Correlation analysis led the authors to outline two distinct pathways of aortic aneurysm formation in SLE. Thoracic aortic aneurysms were not linked with atherosclerosis but were positively correlated with vasculitis, cystic medial degeneration, dissection, and higher mortality rate. Abdominal aortic aneurysms on the other hand showed positive correlation with atherosclerosis associated with prolonged steroid treatment and with better prognosis [2].

Aortitis, the pathologic term for inflammation of the aorta, is broadly subdivided into noninfectious and infectious aortitis. The majority of aortitis cases are noninfectious and include large-vessel vasculitides and other rheumatologic conditions. Infectious aortitis are less common [8]. A normal aorta is normally not prone to infection. However, damage to the aortic wall, such as in cases of atherosclerotic disease, aneurysm, cystic medial degeneration, endothelial damage from diabetes, medical devices, or surgery, makes it weak and vulnerable to infection. Microorganisms can seed hematogenously via the vasa vasorum, contiguously from the adjacent infected tissues, or by traumatic or iatrogenic means. Commonly implicated pathogens include *Salmonella*, *Staphylococcal*, and *Streptococcus* species. Tuberculous aortitis is infrequent in the developed countries [7, 8]. However, due to the high prevalence of tuberculous infections in our country, it is still relevant to include a tuberculous etiology in the differentials. According to a cross-sectional study in 2012, *Staphylococcus aureus* and *Salmonella* species are the most common pathogens (40%), with *Salmonella* as the most prevalent in abdominal aortitis, native aorta, and mycotic aneurysms [9]. Majority of adult cases inflicted with nontyphoid salmonellosis are group B (38%), then followed by group D (30%) and group C (23%) [10]. The known risk factors for invasive *Salmonella* infections are achlorhydria (i.e., intake of proton pump inhibitors) and immunosuppression (i.e., chronic steroids, immunocompromised host, and HIV infection). A minimum of three weeks post source control up to 6-12 weeks is recommended for endovascular infection. And chronic suppressive therapy is advised for patients with infected prosthetic materials, biliary tract abnormalities, and people living with HIV with nontyphoidal bacteremia, low CD4 counts, or those poorly responding to antiretroviral therapy. TMP-SMX 160/800 mg OD, levofloxacin 500 mg OD, or ciprofloxacin 500-750 mg OD can be given indefinitely [11].

In our patient's case, evidence of inflammation was demonstrated in the histopathologic sections of the excised aorta. The infiltrates were predominantly neutrophilic with foam cells thereby favoring an atherosclerotic over a vasculitic pathophysiology of the aneurysm. This is in contrast with the primarily lymphocytic inflammatory pattern of SLE vasculitis [12]. We can therefore surmise that our patient's chronic SLE, coupled with the long-term steroid use, contributed to accelerated atherosclerosis and eventual aneurysm formation. This became a nidus for bacterial infiltration and proliferation, which in turn, may have further perpetuated aneurysmal growth and dissection. We hypothesize that the initial diarrhea of our patient may be due to inadequately treated *Salmonella*, with bacterial seeding on the abdominal aneurysm causing aortitis and the abscess formation. SLE patients have increased susceptibility to *Salmonella* presumably due to defective lymphocyte function, impaired effector cells (e.g., macrophage, monocytes, and granulocytes), and depletion of C3 opsonizing activity [13]. Our patient did not present with classic signs and symptoms of infection because she has been chronically maintained on multiple immunosuppressive agents to control SLE activity. The open repair allowed for gross inspection and discovery of the small pocket of abscess which was missed on the CT aortogram. It also allowed us to obtain biopsies and better understand the underlying disease process of aneurysm formation.

## 4. Conclusion

Aortic complications are uncommon manifestations of SLE. These can be caused by vasculitis, atherosclerosis, infection, or a combination of these mechanisms. We highlight the significance of having a high index of suspicion for infectious causes of aortitis among immunocompromised patients presenting with aneurysm prior to pursuing an endovascular versus an open approach for repair. Inadvertently placing an endovascular graft in an infected aortic aneurysm would have led to graft infection and catastrophic morbidity.
